# Co-Administration of Resolvin D1 and Peripheral Nerve-Derived Stem Cell Spheroids as a Therapeutic Strategy in a Rat Model of Spinal Cord Injury

**DOI:** 10.3390/ijms241310971

**Published:** 2023-06-30

**Authors:** Seung-Young Jeong, Hye-Lan Lee, SungWon Wee, HyeYeong Lee, GwangYong Hwang, SaeYeon Hwang, SolLip Yoon, Young-Il Yang, Inbo Han, Keung-Nyun Kim

**Affiliations:** 1 Spine & Spinal Cord Institute, Department of Neurosurgery, College of Medicine, Yonsei University, Seoul 03722, Republic of Korea; spinejeong@gmail.com (S.-Y.J.); hllee4@yuhs.ac (H.-L.L.); stephwee95@yuhs.ac (S.W.); kimura416@yuhs.ac (H.L.); hwang32@yuhs.ac (G.H.); sayoon@yuhs.ac (S.H.); goodiysl@yuhs.ac (S.Y.); 2 Graduate Program in Bioindustrial Engineering, Yonsei University, Seoul 03722, Republic of Korea; 3 Paik Imje Memorial Institute for Clinical Research, InJe University College of Medicine, Busan 47392, Republic of Korea; pathyang@paik.ac.kr; 4 Department of Neurosurgery, CHA University School of Medicine, CHA Bundang Medical Center, Seongnam-si 13496, Republic of Korea

**Keywords:** spinal cord injury, regeneration, anti-inflammation, peripheral nerve-derived stem cell spheroid, resolvin D1

## Abstract

Spinal cord injury (SCI), primarily caused by trauma, leads to permanent and lasting loss of motor, sensory, and autonomic functions. Current therapeutic strategies are focused on mitigating secondary injury, a crucial aspect of SCI pathophysiology. Among these strategies, stem cell therapy has shown considerable therapeutic potential. This study builds on our previous work, which demonstrated the functional recovery and neuronal regeneration capabilities of peripheral nerve-derived stem cell (PNSC) spheroids, which are akin to neural crest stem cells, in SCI models. However, the limited anti-inflammatory capacity of PNSC spheroids necessitates a combined therapeutic approach. As a result, we investigated the potential of co-administering resolvin D1 (RvD1), known for its anti-inflammatory and neuroprotective properties, with PNSC spheroids. In vitro analysis confirmed RvD1’s anti-inflammatory activity and its inhibitory effect on pro-inflammatory cytokines. In vivo studies involving a rat SCI model demonstrated that combined therapy of RvD1 and PNSC spheroids outperformed monotherapies, exhibiting enhanced neuronal regeneration and anti-inflammatory effects as validated through behavior tests, quantitative reverse transcription polymerase chain reaction, and immunohistochemistry. Thus, our findings suggest that the combined application of RvD1 and PNSC spheroids may represent a novel therapeutic approach for SCI management.

## 1. Introduction

Spinal cord injury (SCI), primarily caused by trauma, results in lasting and irreversible impairments especially impacting motor, sensory, and autonomic functions. The high prevalence of SCI in younger populations presents complex challenges that span social, economic, and medical dimensions. National epidemiological surveys suggest an incidence of 40 to 80 cases per million people per year [1,2,3,4].

SCI is commonly divided into primary and secondary injuries. Primary injuries refer to the direct damage to nerve tissue and blood vessels caused by physical trauma, while secondary injuries involve a series of pathophysiological processes, such as inflammation, reactive oxygen species formation, ischemia, peroxidation, edema, and cell death, which result from the primary injury [5,6,7,8,9,10,11,12,13,14,15,16,17]. Currently, the only recognized therapeutic interventions are high-dose corticosteroid administration within the first 24 h post-injury, surgical decompression, and spinal stabilization [18,19,20,21]. However, there is a notable lack of effective treatment options to prevent or mitigate secondary damage following primary injury or to promote regeneration of the damaged spinal cord. The limited effectiveness and potential adverse effects of conventional therapies highlight the need for innovative treatment strategies [22,23]. In this regard, stem cells, which are known for their neuroregenerative and neuroprotective properties, have emerged as promising tools for managing SCI.

Following SCI, the spinal parenchyma becomes infiltrated by neutrophils, macrophages, T cells, and microglia, which release pro-inflammatory cytokines, such as tumor necrosis factor alpha (TNF-α), interleukin-1 beta (IL-1β), interleukin-1 alpha (IL-1α), and interleukin-6 (IL-6) [24,25,26,27]. This neuroinflammation triggers the formation of necrotic cavities surrounded by glial scars, hindering SCI progression [27,28,29]. Simultaneously, activated macrophages and microglia participate in phagocytosing necrotic and damaged tissues, a crucial process for promoting a nerve regeneration-friendly environment [30,31].

The focus of current research was to investigate various stem cell sources that could facilitate recovery from SCI. Stem cell therapy, which has shown potential protective properties, has been extensively studied in different SCI models. Importantly, the regenerative capabilities of various stem cells, such as bone marrow-derived mesenchymal stem cells (BM-MSCs), cord blood (CB)-derived MSCs, adipose tissue (AT)-derived MSCs, neural stem cells (NSCs), and embryonic cell-derived oligodendrocyte progenitor cells (OPCs), are being assessed for potential use in SCI treatment [32,33,34]. In our previous work, we successfully isolated, cultured, and identified peripheral nerve-derived stem cells (PNSCs) from adult peripheral nerves. These PNSCs, which exhibit characteristics similar to those of neural crest stem cells (NCSCs), can differentiate into cells of both nervous and mesodermal lineages, and they express and secrete neurotrophic and growth factors, including nestin, p75, Sox10, CD105, P0, S100β, CD31, and CD45 [4]. They have been employed as stem cell therapies for SCI regeneration, particularly due to their ability to secrete various neurotrophic factors, including glial cell line-derived neurotrophic factor, insulin-like growth factor, nerve growth factor, and neurotrophin-3. We also observed enhanced PNSC functionality when they were developed into spheroids, which secrete neurotrophic factors in amounts several-fold greater than non-spheroid PNSCs and express various types of extracellular matrices. Our previous study validated the effectiveness of PNSC spheroid transplantation in functional recovery and neuronal regeneration in a rat model of SCI [4]. However, the challenge of mitigating inflammation associated with secondary injury remains, necessitating further therapeutic interventions.

Numerous studies have aimed to reduce inflammation following SCI. Recent research has demonstrated the effectiveness of intrathecal injection of resolvin D3 (RvD3) in promoting the resolution of inflammation, neuroprotection, functional neurological recovery, and decreased thermal hyperalgesia after SCI [35]. Beneficial outcomes include the downregulation of pro-inflammatory cytokines, the conversion of the macrophage phenotype to M2, the preservation of tight junctions in the blood–spinal cord barrier, neuroprotection, and the prevention of acute-phase glial/fibrotic scar formation [36,37,38].

In a mouse model of brain injury, resolvin D1 (RvD1) administration has been shown to provide neuroprotection, reduce inflammatory responses, and promote functional recovery. RvD1 is known to exert its neuroprotective effects via the ALX/FPR2 receptor and by regulating microRNAs primarily affecting neuroinflammation [39,40]. However, the impact of RvD1 on SCI remains unexplored. Considering its established anti-inflammatory and neuroprotective properties [39,41,42], RvD1 may enhance the viability of PNSC spheroids and improve their regenerative function. Furthermore, the combined application of RvD1 and PNSC spheroids may potentiate a synergistic effect on the restoration of neurological function in a rat model of SCI.

## 2. Results

### 2.1. In Vitro Anti-Inflammatory Effects of RvD1

**Figure 1:**
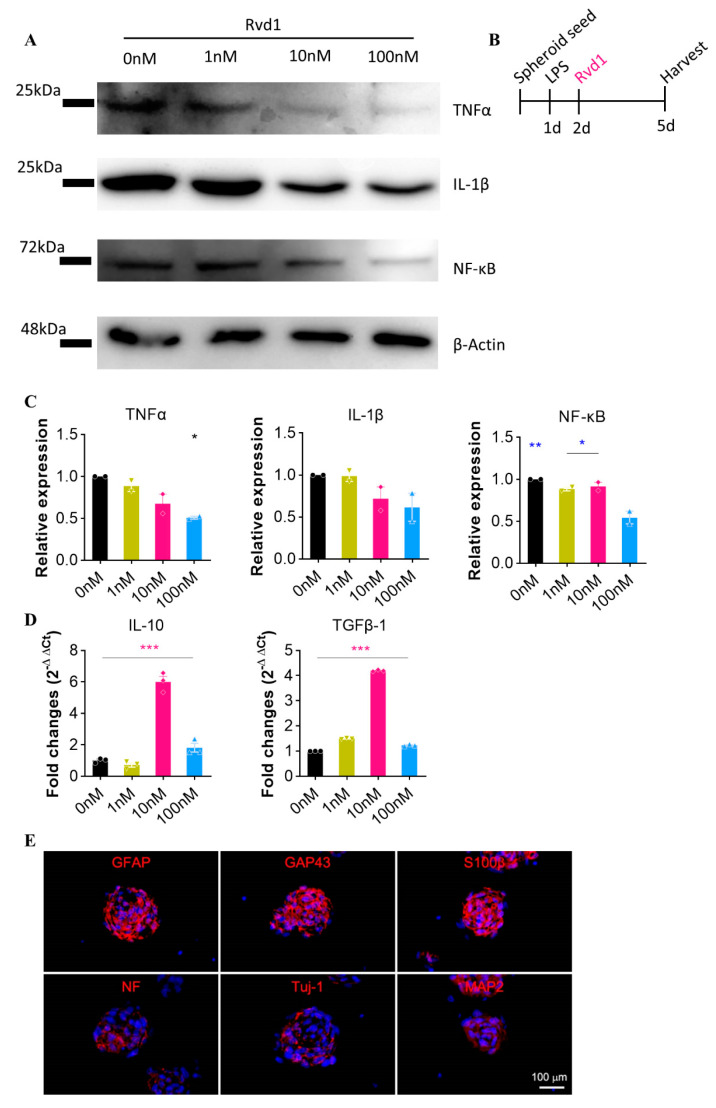
RvD1 demonstrates significant anti-inflammatory properties under in vitro conditions. (**A**) Western blot analysis confirmed the expression of pro-inflammatory cytokines (TNF-α, IL-1β, and NF-κB) in response to RvD1 treatment at concentrations of 0 nM, 1 nM, 10 nM, and 100 nM (*n* = 2). (**B**) Schematic diagram of experiments. (**C**) Quantitative data from Western blot. Expression level is normalized with the 0 nM group. (**D**) The expression of the anti-inflammatory cytokines IL-10 and TGF-β, as assessed by qRT-PCR, following RvD1 treatment at concentrations of 0 nM, 1 nM, 10 nM, and 100 nM. (**E**) Spheroid PNSCs expressed markers for committed glial cells (GFAP, S100β and GAP43) and neuronal cells (neurofilament; NF, Tuj-1, and MAP2). Blue means DAPI. * *p* < 0.05, ** *p* < 0.01, and *** *p* < 0.001.

Following the initiation of spheroid seeding under in vitro conditions, inflammation was artificially induced by lipopolysaccharide (LPS) stimulation, which led to the identification of pro-inflammatory cytokines. To normalize the analysis, β-actin was used as the housekeeping gene. In the Western blot analysis, RvD1 was associated with a relative decrease in pro-inflammatory cytokines, such as TNF-α, IL-1β, and nuclear factor kappa-light-chain-enhancer of activated B cells (NF-κB) at concentrations of 0 nM, 1 nM, 10 nM, and 100 nM compared to β-actin (Figure 1A,C). Furthermore, the expression levels of anti-inflammatory neurotrophic factors, specifically interleukin-10 (IL-10) and transforming growth factor beta (TGF-β), which are secreted by RvD1, were also assessed using qRT-PCR. The presence of these anti-inflammatory cytokines was confirmed in the RvD1-treated cells, with significant upregulation of expression levels observed at 10 nM (Figure 1D). Overall, our data support the idea that RvD1 exhibits an anti-inflammatory effect by reducing the secretion of pro-inflammatory cytokines in an in vitro setting.

### 2.2. Combined Therapy for Functional Recovery and Tissue Regeneration

To investigate the therapeutic potential of PNSC spheroids and RvD1, a compressive SCI rat model was employed. One week after injury, PNSC spheroids were directly administered as single-cell spheroids. These PNSC spheroids, which exhibit stability, immune expression properties similar to NCSCs, and the ability to differentiate into neuronal and mesenchymal cells, continuously secrete various neurotrophic and anti-inflammatory factors. We tested their potency for differentiation into neuroglial cells by employing a 3D spheroid culture system that enables the activation of the endogenous canonical Wnt pathway signaling and the intrinsic differentiation cascades [1]. PNSCs, when seeded on culture dishes that prevent cell attachment, aggregated with each other and assembled spontaneously into multicellular spheroids within 1 d of culture. Under the spheroid culture conditions, PNSC spheroids expressed glial cell-related markers, including GFAP, GAP43, and S100b, without any requirement for differentiation inducers. At 7 d of culture, more than 80% of the PNSCs expressed GFAP (83.7 ± 6.5%), GAP43 (89.5% ± 8.2%), and S100b (84.9 ± 7.3%), indicative of the PNSCs showing an innate propensity to differentiate in vitro into glial cell lineage without a requirement for differentiation inducers. By inducing cells with neurogenic differentiation inducers, such as NGF, BDNF, and cAMP, PNSCs expressed neural cell markers, including Tuj1+ (15.4 ± 6.8%), MAP2 (1.1 ± 2.7%), and NF200 (26.3 ± 3.4%), at 7 d of culture (Figure 1E), indicating that PNSCs have the ability to differentiate to neural cells but preferentially differentiate into glial cells, thus demonstrating therapeutic potential in SCI models. 

**Figure 2:**
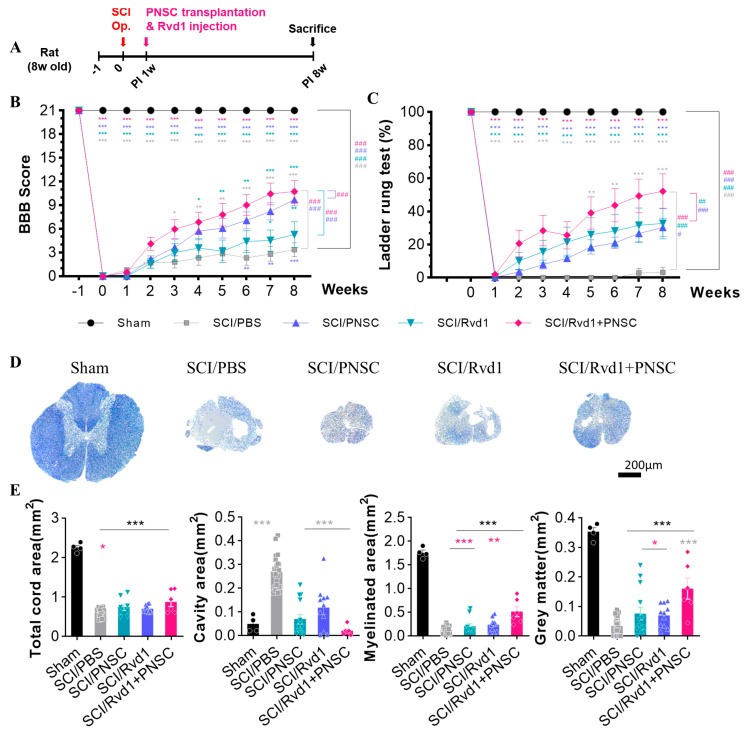
Functional restoration and tissue regeneration following combined therapy (**A**) Schematic diagram of experiments. (**B**) BBB scores post-SCI for all groups (sham, SCI/PBS, SCI/RvD1, SCI/PNSC, SCI/RvD1+PNSC), from 1 week pre-injury to 8 weeks post-injury. (**C**) Ladder rung test results post-SCI for all groups from the day of injury to 8 weeks later. (**D**) Eriochrome cyanine–cresyl violet staining of spinal cord cross-sections for myelin in all groups (sham, n = 3; SCI/PBS, n = 4; SCI/RvD1, n = 6; SCI/PNSC, n = 6; and SCI/RvD1+PNSC, n = 6) for the assessment of tissue regeneration at 4 and 8 weeks post-SCI. (**E**) Evaluation of total cord area, cavity area, myelinated area, and gray matter area from eriochrome cyanine staining across all groups. All data were processed using two-way analysis of variance with Tukey’s multiple comparisons test. The *p*-values are indicated as follows: * *p* < 0.05, ** *p* < 0.01, and *** *p* < 0.001 And analysis by linear mixed effects model is expressed to ^#^ *p* < 0.05, ^##^ *p* < 0.01, and ^###^ *p* < 0.001.

Open field tests were performed weekly, and motor function was assessed at the 8-week mark using the Basso, Beattie, and Bresnahan (BBB) scale. Following SCI, the SCI/phosphate-buffered saline (PBS) group showed a significant decline in motor function. Although the SCI/RvD1 group exhibited minor behavioral improvement, the change was not significant. In contrast, the SCI/PNSC group demonstrated a gradual and significant increase in the BBB score, with the most notable enhancement observed in the SCI/RvD1+PNSC combination group. At 8 weeks post-injury, the average BBB score for the SCI/RvD1+PNSC group was 10.75 ± 1.38, indicative of hind limb weight-supported walking (Figure 2B). These results suggest that RvD1 may enhance the effects of PNSCs.

A ladder rung test was utilized to qualitatively assess locomotion and limb coordination. Video recordings were carefully examined on a frame-by-frame basis. Measurements were taken weekly over an 8-week period following SCI for all groups. Two independent observers determined the ratio of accurately placed steps to total steps, converting the score into a percentage. In the SCI/PBS group, no functional recovery was observed for the 8 weeks after SCI. However, ladder rung test scores gradually improved in the SCI/RvD1 and SCI/PNSC groups. The greatest increase was observed in the SCI/RvD1+PNSC group, with an average score of 52.5 ± 2.5 at 8 weeks post-injury (Figure 2C). The best outcomes in locomotion and limb coordination assessment were confirmed in the SCI/RvD1+PNSC combination group.

To investigate the relationship between functional recovery and tissue regeneration, spinal cord samples from each group were stained with cresyl violet and eriochrome cyanine (EC) at 4 and 8 weeks post-injury. The sham group showed no evidence of spinal cord degeneration. In contrast, substantial cavitation and tissue contraction were observed in the SCI/PBS group. Tissue shrinkage was also apparent in the spinal cords of the SCI/PNSC and SCI/RvD1 groups, albeit with a reduced, yet still considerable, cavity presence (Figure 2D). Conversely, the SCI/RvD1+PNSC group exhibited an increase in the total cord, myelinated, and gray matter areas, along with a significant reduction in the cavity area compared to the other groups (Figure 2E). This outcome indicated the occurrence of remyelination and the inhibition of demyelination near the injury site. As a result, the spinal cord tissue regeneration in the combination group was similar to that observed in the sham group. Additionally, a well-connected tissue structure was confirmed around the injury site.

These observations corresponded closely to the BBB scores, indicating that PNSC spheroids, when combined with RvD1, can promote spinal cord regeneration and contribute to functional recovery.

### 2.3. Alleviation of Mechanical Allodynia through Combined Therapy

**Figure 3:**
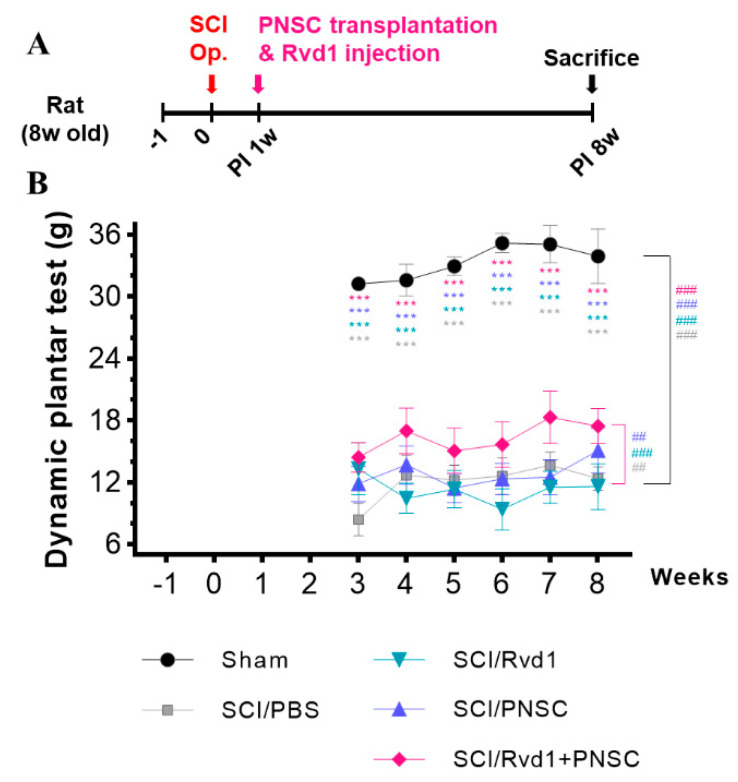
Pain mitigation through combined therapy. (**A**) Schematic diagram of experiments. (**B**) Mechanical allodynia was evaluated using the dynamic plantar test. Testing began 3 weeks after SCI and continued until the 8-week mark. All data were statistically processed using two-way analysis of variance coupled with Tukey’s multiple comparisons test. Multiple comparisons results expressed by *** *p* < 0.001. And analysis by linear mixed effects model is expressed to ^##^ *p* < 0.01, and ^###^ *p* < 0.001.

Mechanical allodynia was assessed using the dynamic plantar test. Evaluations began 3–4 weeks after SCI, when the hind paws of the subjects were oriented downward. In the sham manipulation group, hind paw retraction was observed at an average force of 33.34 g. All groups displayed hypersensitivity to mechanical stimuli 3 weeks post-injury. At 8 weeks post-injury, hypersensitivity persisted in the SCI/PBS, SCI/RvD1, and SCI/PNSC groups, with paw withdrawal noted at 12.4 g, 11.6 g, and 15.1 g, respectively. In contrast, the SCI/RvD1+PNSC group exhibited lower sensitivity to stimuli, withdrawing the paw at 17.46 g at 8 weeks post-injury. In summary, the SCI/RvD1+PNSC group showed an improvement in mechanical allodynia, approaching sham group levels at 8 weeks after SCI (Figure 3).

### 2.4. Outcomes of Immunohistochemical Analysis

**Figure 4:**
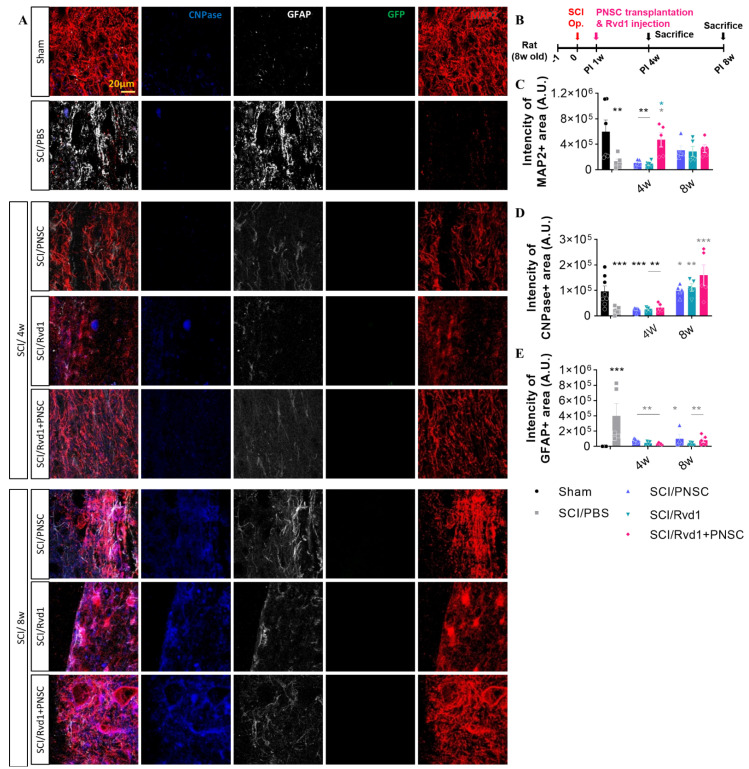
Outcomes of in vivo immunohistochemical analysis. (**A**) Representative images depict staining for CNPase (blue), GFAP (gray), GFP (green), and MAP2 (red) at 4 and 8 weeks following SCI. (**B**) Schematic diagram of experiments. (**C**) Aggregate MAP2 intensity across all study groups (sham, n = 5; SCI/PBS, n = 10; SCI/RvD1, n = 20; SCI/PNSC, n = 20; and SCI/RvD1+PNSC, n = 20). Analysis was conducted using ordinary one-way ANOVA with Tukey’s multiple comparisons test. (**D**) Overall intensity of CNPase staining across all groups (sham, n = 5; SCI/PBS, n = 10; SCI/RvD1, n = 20; SCI/PNSC, n = 20; and SCI/RvD1+PNSC, n = 20). (**E**) Total intensity of GFAP staining across all groups (sham, n = 5; SCI/PBS, n = 10; SCI/RvD1, n = 20; SCI/PNSC, n = 20; and SCI/RvD1+PNSC, n = 20). Analysis was conducted using ordinary one-way ANOVA with Tukey’s multiple comparisons test. All data are presented as the mean ± the standard error of the mean; * *p* < 0.05, ** *p* < 0.01, and *** *p* < 0.001.

In a previous study, we confirmed that PNSC spheroids express neural stem (NS) lineage markers, including nestin, p75NTR, and CD105. Furthermore, neural crest (NC)-specific transcription factors, such as Sox2, Sox9, and Sox10, were preserved in PNSCs, and markers indicative of commitment to the Schwann cell lineage (including GFAP, GAP43, and S100b) were also expressed in PNSC spheroids. In the present investigation, we examined oligodendrocytes, neurons, and astrocytes using antibodies against MAP2, CNPase, and GFAP at 4 and 8 weeks post-injury (Figure 4A).

No differentiated PNSC group was observed, as cells did not maintain viability at 7 weeks post-transplantation. Despite the lack of differentiated cells, significant changes in the expression of neuronal and glial cell markers were observed. MAP2, an early-stage neuronal marker, exhibited the highest expression in the combination therapy group, suggesting the most effective neuronal regeneration (Figure 4C). Although MAP2 expression levels at 8 weeks were slightly lower than those at 4 weeks, further disparities may be revealed with the analysis of cells at later stages. CNPase, an oligodendrocyte marker, showed no significant difference from the SCI/PBS group at 4 weeks. However, at 8 weeks, a considerable increase in expression was observed in the SCI/RvD1, SCI/PNSC, and SCI/RvD1+PNSC groups, suggesting enhanced remyelination compared to the control group (Figure 4D). GFAP, an astrocyte marker, is primarily expressed in reactive astrocytes but not in naive astrocytes. The most extensive glial scars were observed in the PBS group, in which GFAP expression was highest, likely interfering with neuronal regeneration. This finding was supported by the lowest MAP2 expression level in the PBS group. In the RvD1 group, GFAP expression was the lowest; however, MAP2 and CNPase expression levels were also low, indicating that, although glial scar formation was inhibited, it did not adequately promote neuronal regeneration. In the combination group, GFAP expression was relatively low, while MAP2 and CNPase expression levels were the highest, suggesting that glial scar formation was suppressed, and neuronal regeneration was the most pronounced (Figure 4E).

### 2.5. Assessment of In Vivo Cell Survival and Expression of Neurotrophic Factor Following Transplantation

**Figure 5:**
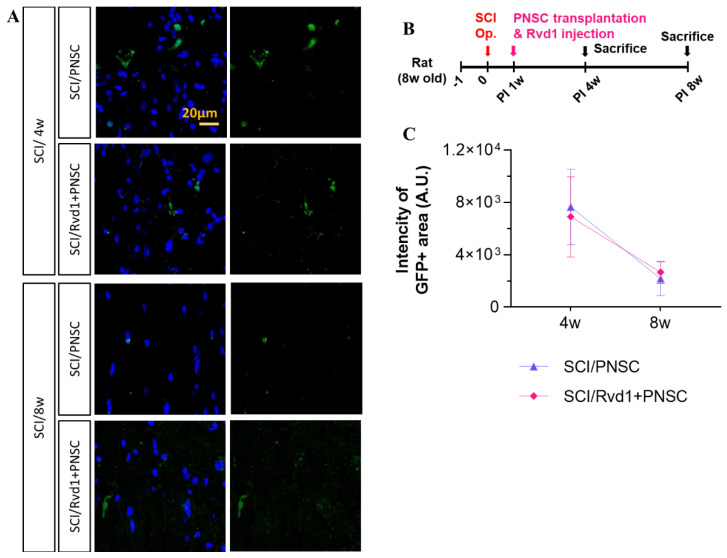
Survival of transplanted cells in an in vivo experiment. Evaluation of transplanted peripheral nerve-derived stem cell (PNSC) and RvD1+PNSC viability. (**A**) Representative confocal images display GFP (white arrow) and DAPI (blue) staining 4 and 8 weeks after injury (sham, n = 5; SCI/PBS, n = 10; SCI/RvD1, n = 20; SCI/PNSC, n = 20; and SCI/RvD1+PNSC, n = 20). (**B**) Schematic diagram of experiments. (**C**) The total intensity of GFP+ cells per mm^2^ is shown for all groups. Data were analyzed using one-way ANOVA followed by Tukey’s post-hoc test for multiple comparisons.

The survival of transplanted cells was evaluated at 4 and 8 weeks post-transplantation. Transplanted PNSC spheroids were identified using GFP, as they were initially isolated from murine stem cells. At 4 weeks after transplantation, only a minimal number of cells were found to have survived in both the SCI/PNSC and SCI/RvD1+PNSC groups. However, at the 8-week mark, a small number of GFP-positive cells were observed in all study groups (Figure 5A,C). This finding suggests that, although the cells within the SCI/PNSC and SCI/RvD1+PNSC groups may not demonstrate long-term survival, they continue to impact the spinal environment through paracrine effects, ultimately contributing to improved functional behavior. 

**Figure 6:**
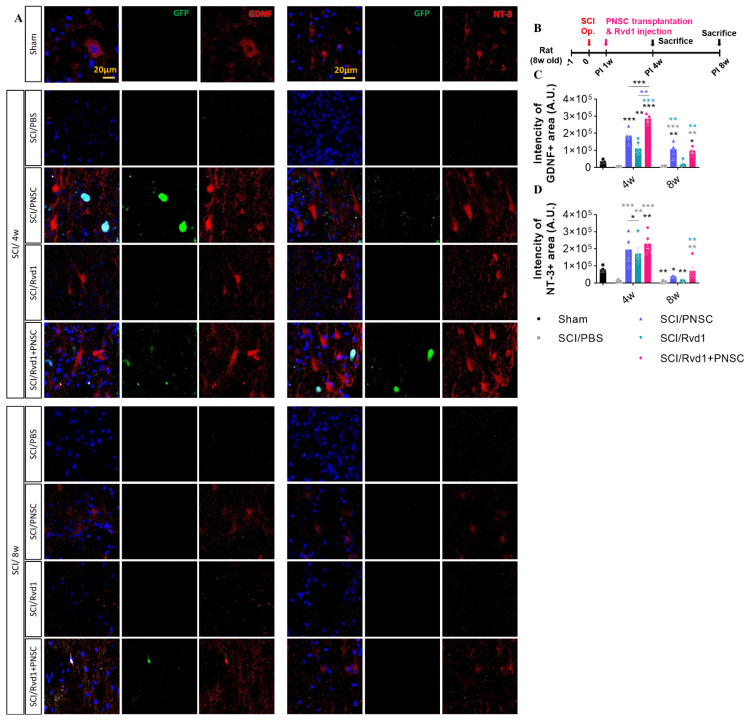
The Rvd1 extended the expression of GDNF and NT-3 after SCI (**A**) Representative confocal imaging, with blue indicating DAPI, green indicating GFP, and red indicating either GDNF or NT-3. (**B**) Schematic diagram of experiments. (**C**) Total intensity of GDNF. (**D**) Total intensity of NT-3. Data were analyzed using one-way ANOVA. Multiple comparisons results expressed by * *p* < 0.05; ** *p* < 0.01; *** *p* < 0.001.

Furthermore, our previous report provided evidence of the expression of various neurotrophic factors, including BDNF, GDNF, IGF, IL-6, NGF, and NT-3, in PNSCs. Notably, we observed increased expression of GDNF and NT-3 in spheroid forms of PNSCs, suggesting that PNSC spheroids have a prolonged and heightened influence on their expression, particularly in the context of SCI. This current study aimed to verify the impact of Rvd1 on the expression of GDNF and NT-3 by PNSC spheroids. Our results indicated a significant increase in the expression of GDNF and NT-3 in all groups, including PNSC spheroids, Rvd1, and Rvd1 combined with PNSC spheroid transplants, at 3 weeks post-transplantation (4 weeks after injury) (Figure 6A–D). Notably, the group receiving Rvd1 and PNSC co-transplantation demonstrated the most substantial increase in GDNF expression compared to the other groups. Furthermore, our previous study already demonstrated the ability of PNSC spheroids to maintain the secretion of neurotrophic factors for a more prolonged period compared to single cells. Confirming these findings, our current study demonstrated that Rvd1 contributes to extended secretion of GDNF and NT-3 compared to the group transplanted with PNSC spheroids alone, with a notable emphasis on the sustained effect on NT-3 at 8 weeks after injury.

### 2.6. In Vivo Anti-Inflammatory Effects of RvD1

**Figure 7:**
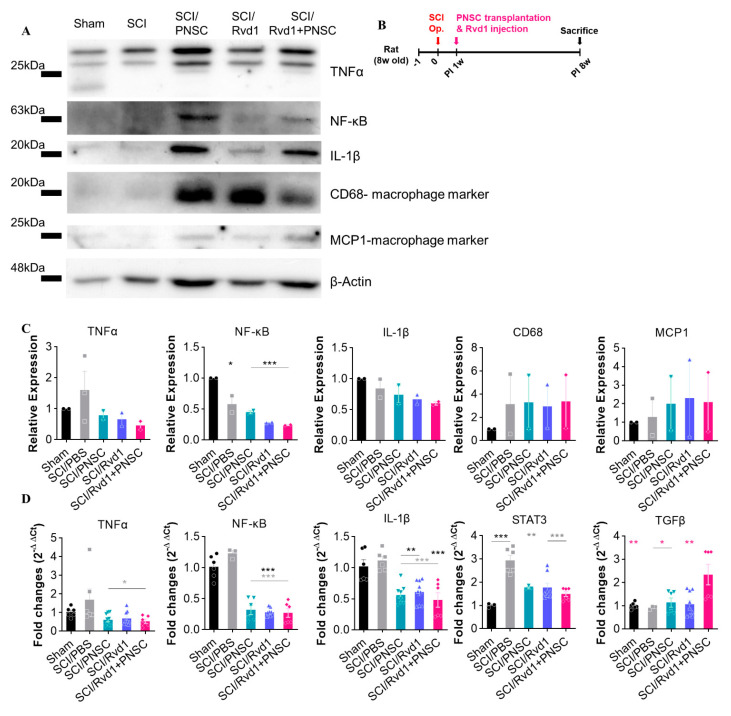
Demonstration of RvD1’s potent anti-inflammatory action in vivo. (**A**) The production of pro-inflammatory cytokines (TNF-a, NF-κβ, IL-1β, and the macrophage markers CD68 and MCP1) was analyzed in all groups (sham, n = 2; SCI/PBS, n = 2; SCI/RvD1, n = 2; SCI/PNSC, n = 2; and SCI/RvD1+PNSC, n = 2) using Western blotting. (**B**) Schematic diagram of experiments. (**C**) Quantitative analysis of Western blot. All data are normalized with the sham group. (**D**) The SCI/RvD1+PNSC group displayed the highest expression of the anti-inflammatory cytokine TGF-β, as determined by qRT-PCR. In contrast, significantly greater expression of pro-inflammatory cytokines (NF-κB, IL-1β, STAT3 and TNF-α) was observed in the SCI/PBS group compared to the three other groups. Data were analyzed using one-way ANOVA. Multiple comparisons results expressed by * *p* < 0.05; ** *p* < 0.01; *** *p* < 0.001.

Following spheroid implantation, five groups (sham, SCI, SCI/PNSC, SCI/RvD1, and SCI/RvD1+PNSC) were assessed for inflammatory markers using Western blotting. Consistent with our in vitro findings, we observed a significant increase in pro-inflammatory cytokines (TNF-a, IL-1β, and NF-κB) after SCI. Furthermore, suppressing the secretion of pro-inflammatory cytokines proved difficult with PNSC transplantation alone. RvD1, which has anti-inflammatory properties, showed some effectiveness in reducing the expression of pro-inflammatory markers, as well as the recruitment of the CD68 macrophage marker and the MCP1-macrophage marker. However, the most potent effect was seen with the combination of RvD1 and PNSC, which also led to a decrease in overall macrophage recruitment (Figure 7A,C). 

The expression of the anti-inflammatory cytokine TGF-β and pro-inflammatory cytokines NF-κB, IL-1β, and TNF-a across all groups was analyzed using qRT-PCR. A significantly higher level of TGF-β was observed in the SCI/RvD1+PNSC group, while no differences were detected among the other groups. The expression of pro-inflammatory cytokines (NF-κB, IL-1β, STAT3, and TNF-a) was considerably higher in the SCI/PBS and sham groups compared to the SCI/PNSC, SCI/RvD1, and SCI/RvD1+PNSC groups. However, no statistically significant differences were found among these three groups (Figure 7D). 

## 3. Discussion

Transplanting cells into challenging microenvironments, such as those characterized by excessive inflammatory responses, free radicals, and hypoxia following SCI, necessitates the advancement of cell therapy or cell transplantation technologies to enhance the cell survival rate and functionality [29,43,44,45,46,47]. In an effort to develop a novel technology for PNSC isolation and culture, as well as a culture system in which NCSCs can spontaneously form three-dimensional (3D) microspheres in a suspension culture environment, we developed a 3D organ culture system that mimics tissue homeostasis from the peripheral nerves of brain-dead patients [4]. SCI regeneration was achieved by the secretion of factors including brain-derived neurotrophic factor, glial-derived neurotrophic factor (GDNF), insulin-like growth factor (IGF), IL-6, nerve growth factor, and neurotrophin-3 (NT-3).

We hypothesized that the combination of RvD1 and PNSC spheroids promotes myelin regeneration and functional recovery in SCI by regulating the inflammatory response and promoting angiogenesis, compared to a single treatment modality. In this study, we evaluated the impact of the combination of RvD1 and PNSC spheroids on spinal cord regeneration in vitro and in a rat model of SCI.

Various stem cell components, including BM-MSCs, CB-MSCs, AT-MSCs, NSCs, and OPCs, have been employed as primary stem cell treatments for SCI, with numerous clinical studies conducted [48,49,50,51]. BM-MSCs are multipotent adult stem cells derived from the bone marrow stroma, contributing to hematopoiesis and bone regeneration. They can be directly transplanted into the damaged spinal cord or administered intravenously due to their homing properties [49,52,53]. CB-MSCs, obtained from blood or the umbilical cord, have exhibited a promising profile of neurotrophic, anti-apoptotic, and anti-inflammatory effects in an animal model of SCI [54,55,56]. Yao et al. reported autonomic restoration and alterations in somatosensory evoked potentials following intravenous and intrathecal infusion of human umbilical cord blood stem cells in patients with traumatic SCI [57]. AT-MSCs are harvested from adherent cultures of the stromal–vascular fraction isolated from fatty tissues and share similar characteristics with BM-MSCs [58,59,60]. This cell type exhibits the capability for multilineage differentiation, transforming into adipocytes, osteocytes, chondrocytes, smooth muscle cells, hepatocytes, and neurons [61,62,63]. NSCs, originating from the subventricular zone of the hippocampus and a segment of the central canal of the spinal cord, are characterized by their multipotency and self-renewal capabilities [64]. These NSCs have the potential to differentiate into distinct neuronal or glial lineages. Multiple studies have highlighted the success of functional recovery following NCSC transplantation in SCI models. They have demonstrated the capacity to restore lost neuronal and glial tissues, providing essential trophic support [65,66,67].

NCSCs are specialized cells that emanate from the neural tube during embryogenesis and migrate to various organs and tissues throughout the body, contributing to the formation of tissues and cells in the peripheral nervous system. Although these cells were traditionally thought to disappear after birth, recent research has confirmed the presence of NCSC-like cells in various adult tissues, including the bone marrow, skin, teeth, olfactory nerves, cornea, gastrointestinal tract, palate, and carotid artery [58,68,69,70]. Once isolated, cultured, and identified, these adult-derived NCSC-like stem cells possess the unique ability to differentiate into neurons and Schwann cells, providing potential therapeutic strategies for SCI due to their neuroactive secretion capabilities.

Prominent sources of NCSC-derived cells include skin-derived precursors and olfactory ensheathing cells (OECs), which are currently being investigated as potential treatments for SCI [71,72,73,74]. However, practical challenges exist in generating sufficient therapeutic quantities from skin or olfactory nerves. Notably, complications such as cyst formation post-transplantation due to nasal mucosal cell contamination have been reported in the case of OECs, highlighting the need for stringent safety measures.

In the context of peripheral nerve injury, the cells responsible for tissue regeneration produce Schwann cells with a repair phenotype through de-differentiation or reprogramming mechanisms. These reparative Schwann cells secrete neuroactive and anti-inflammatory factors that are crucial for peripheral nerve regeneration. It has been demonstrated that these regenerated Schwann cells share similarities with Schwann progenitor cells or NCSCs in terms of their characteristics. 

In our previous study, we successfully isolated PNSCs that exhibited characteristics similar to those of NCSCs using a 3D culture technique. PNSCs were found to express NC-specific markers, including nestin, p75NTR, Sox10, and CD105, as confirmed by flow cytometry assessments. Furthermore, tissue staining revealed their induction into mesoderm-lineage cells, specifically adipocytes and osteocytes [4]. Based on these findings, it can be concluded that PNSCs have the capacity to differentiate into both neuronal and mesenchymal cells, and they release neurotrophic and anti-inflammatory factors. The functionality of PNSCs is rooted in their paracrine effects, which are driven by the expression of neurotrophic factors. Two such factors, GDNF and NT-3, are consistently expressed at high levels in PNSCs and are known for their neuroprotective effects and ability to stimulate neuronal regeneration. This study provides evidence of significant upregulation of GDNF and NT-3 expression in both the PNSC and Rvd1 transplanted groups. Notably, the PNSC-transplanted group exhibited higher and sustained expression of GDNF and NT-3 compared to the Rvd1 group [4]. Additionally, the combined transplantation of Rvd1 and PNSCs demonstrated a synergistic effect on the expression of these neurotrophic factors, which can be attributed to the anti-inflammatory properties of Rvd1. The anti-inflammatory effect of Rvd1 is well established in the literature, and several studies have reported that anti-inflammatory drugs can induce an increase in the production of neurotrophic factors [75,76,77]. Consequently, the administration of Rvd1 enhances the paracrine effect of PNSC spheroids, potentially leading to more effective functional recovery in the context of SCI. PNSCs have shown potential in axon regeneration and remyelination. However, axonal growth beyond the graft is believed to be limited due to glial cell scarring.

Following SCI, a marked inflammatory response typically occurs, with resident microglia and macrophages as the primary actors. Their selective regulation is crucial for disease progression. The pro-inflammatory environment attracts numerous peripheral monocytes/macrophages with varying phenotypes. Some, such as pro-inflammatory M1 macrophages, may cause harmful effects by exacerbating neurodegeneration and tissue loss, while others, such as M2 macrophages, promote neuroprotection and regeneration [33,36,37,38,78].

In this study, we determined that PNSC spheroids contributed to functional recovery and reparative effects in an SCI model. However, controlling inflammation, which is a key factor in secondary injury, proved difficult with the sole use of PNSC spheroids. As a result, alternative neuroprotective treatments have been investigated. 

Within the realm of SCI, neuroprotective agents are commonly used in clinical settings. These agents can be broadly categorized into calcium antagonists, glutamate release inhibitors, neurotrophic factors, free radical scavengers, cell membrane stabilizers, anti-inflammatory factors, glutamate antagonists, excitatory amino acid antagonists, gamma-aminobutyric acid receptor agonists, and leukocyte adhesion inhibition agents, based on their respective mechanisms of action [9,79,80]. Unfortunately, most of these pharmaceuticals are either ineffective or cause adverse effects. Since much of the neurological damage results from subsequent inflammation, even reducing inflammation could provide beneficial effects for neuroprotection. As mentioned in the introduction, we have been investigating the role of RvD1 among various neuroprotective substances. 

Recent research has increasingly supported the idea that the resolution of acute inflammation is an active process, facilitated by the production of specialized pro-resolving mediators (SPMs). These mediators include resolvins, protectins/neuroprotectins, and maresins. D-series resolvins (such as RvD1 and RvD2) and E-series resolvins (such as RvE1) are derived from the omega-3 polyunsaturated fatty acids docosahexaenoic acid and eicosapentaenoic acid, respectively. They have exhibited significant anti-inflammatory and pro-resolution effects in numerous animal models of inflammation and infection [40,81,82,83]. SPMs modulate inflammation and its resolution via the activation of various cell surface G protein-coupled receptors, which rapidly transmit signals and initiate intracellular pathways to regulate numerous biological functions [84]. Considering that the inflammatory response is a major component of the secondary injury process following SCI, SPMs could potentially modulate inflammation caused by SCI. 

One investigation revealed that the RvD group exhibited anti-inflammatory properties and promoted nerve regeneration. While RvD3 and RvD4 did not influence postoperative pain relief following bone fractures, RvD1, RvD2, and RvD5 effectively reduced mechanical allodynia [85,86,87]. Previously, Kim et al. reported that intrathecal injection of RvD3 effectively promoted inflammatory resolution, neuroprotection, and functional nerve recovery while reducing thermal hyperalgesia in an SCI model [35].

RvD1, which is known for its potent anti-inflammatory effects, has been demonstrated to promote inflammation resolution in numerous studies [88,89]. In a dose-dependent fashion (10–500 nM), it reduced the secretion of pro-inflammatory cytokines, such as IL-6, IL-6Ra, MCP-1, and leptin [88]. RvD1 is recognized for attenuating the gene expression of pro-inflammatory molecules by inducing M2 macrophage polarization in chronic inflammatory autoimmune diseases [90] and acute respiratory distress syndrome, which are characterized by uncontrolled inflammation [91]. Bisicchia et al. reported that RvD1 activated the receptor ALX/FPR2 to coordinate a novel resolution circuit that specifically regulates miR-219a-1-3p and miR-146b, their downstream targets (TLR4 and IL6R), and, to a lesser extent, NF-κβ and CD200 [39].

In our study, we found that RvD1 induced a decrease in pro-inflammatory cytokines (such as TNF-α, IL-1β, and NF-κB) and promoted the release of anti-inflammatory cytokines (such as TGF-β) in the SCI model. Other research has shown that aspirin-triggered (AT)-RvD1 treatment decreased leukocyte recruitment, reduced neutrophil-platelet interactions, and downregulated pro-inflammatory mediators (including IL-1β, IL-6, IL-8, and TNF-α) [40,90]. This outcome may be related to the rapid recognition of necroptotic cells by RvD1-stimulated macrophages.

Following an SCI, 80% of patients experience neuropathic pain in the acute phase, and 30–50% do so in the chronic phase. Secondary damage due to SCI often results in demyelination, which subsequently triggers hyperalgesia and allodynia as consequences of C-fiber hyperexcitability. A study by Lee et al. showed that injecting PNSC spheroids into rats with simulated SCI led to increased NT-3 expression and promoted a remyelination effect, thereby alleviating neuropathic pain [4]. In our study, we found that the combination of PNSC spheroids and RvD1 reduced hyperalgesia and allodynia while improving motor function.

To assess the influence of RvD1 on PNSC functional recovery and neuroregeneration following SCI, we utilized BBB scoring. Our results confirmed that RvD1 enhances the effect on PNSCs. Additionally, we examined allodynia, a common type of neurogenic pain often associated with SCI. Eight weeks after SCI, the combination of RvD1 and PNSCs resulted in the greatest decrease in mechanical allodynia compared to treatments with RvD1 or PNSCs alone. We attribute this superior effect to the combination therapy, which promotes remyelination and reduces demyelination through its anti-inflammatory properties. 

In this study, we examined the effects of co-administering RvD1 and PNSC spheroids in a rat model of SCI. Our findings revealed several significant outcomes, including functional recovery, neuronal regeneration, and reduced mechanical allodynia, demonstrating greater therapeutic benefits compared to other monotherapy strategies in a rat model. It is hypothesized that these effects are due to the enhanced paracrine action of stem cells, as co-administration therapy both in vivo and in vitro has been shown to suppress inflammation following SCI and increase the secretion of various neurotrophic factors. However, it is essential to recognize certain limitations and inconsistencies observed during this study. Although RvD1 was administered once at 1 week post-SCI to achieve an anti-inflammatory effect, further research is needed to evaluate the impact of multiple injections. Moreover, this study was conducted using rats, and insufficient long-term data currently exist regarding the applicability to humans and the potential oncogenic effects associated with the use of stem cells, representing the limitations of this study.

## 4. Materials and Methods

### 4.1. PNSC Two-Dimensional Culture Protocol

In this study, the peripheral nerves (PNs) used for cell isolation were obtained from the common iliac nerve segments of rat donors, following a protocol reviewed and approved by the Institutional Review Board of Inje University Busan Paik Hospital. The epineurium and surrounding connective tissue were carefully removed under a stereomicroscope, and the PN segments were finely chopped into 2- to 3-mm-long pieces using a razor blade. These fragments were then washed with PBS (Cytiva, Marlborough, MA, USA) and suspended in a pre-chilled 0.25% neutral collagen solution. The resulting suspension was transferred to a 100-mm tissue culture dish and incubated at 37 °C in a humidified chamber for 2 h, allowing for the formation of a neutral collagen hydrogel for 3D encapsulation of the PN fragments.

Organ cultures were cultivated under dynamic conditions using an orbital shaker set at 25 rpm for 2 weeks, with the organ culture medium refreshed three times weekly. After 14 days of organ culture, the collagen hydrogels containing PN fragments and outgrown PNSCs were treated with 0.01% collagenase type I to degrade them. Following degradation, the migrated/outgrown PNSCs released from the hydrogels were collected, centrifuged, and resuspended in cell culture medium. The harvested PNSCs were then seeded in cell culture dishes and allowed to proliferate under standard monolayer culture conditions. The growth culture medium used for proliferation consisted of low-glucose Dulbecco modified Eagle medium (DMEM) supplemented with F12, 10% fetal bovine serum, 1% penicillin/streptomycin, 10 ng/mL IGF (Peprotech, Seoul, Republic of Korea), 10 ng/mL epidermal growth factor (Peprotech), and 2 ng/mL basic fibroblast growth factor (Peprotech). Upon reaching 80% confluence in the 150π culture dish, the PNSCs were subcultured to the subsequent passage and detached using 0.5% trypsin–EDTA treatment.

### 4.2. Three-Dimensional Sphere-Like Cell Culture and Differentiation Protocol

Upon reaching 80% confluence, the PNSCs were harvested using 0.5% trypsin–EDTA treatment and resuspended in a specialized suspension culture medium consisting of DMEM enriched with bovine serum, dimethyl sulfoxide, and penicillin/streptomycin. The cultured PNSCs from 150π cell culture dishes were then seeded in a 100π ultra-low attachment cell culture dish (SPL3DTM Cell Floater). The growth of the PNSCs was monitored, and based on their proliferation status, the 3D PNSC spheroids were cultured for a duration ranging from 24 to 72 h. Throughout the entire experimental process, PNSCs from passages 23–24 were used. In this study, we used higher passages than in our previous study, but there was no significant differentiation between lower- and higher-passage cells in doubling time and differentiation ability (Figure 1E) [4]. 

The ability of isolated PNSCs to differentiate into neuroglial cells was tested by seeding 1 × 10^3^ cells on a 96-well spheroid microplate (4520, Corning, NY, USA) and adding 100 mL of an glial differentiation medium consisting of DMEM/F12 medium supplemented with 1% calf serum (CS), 1 μM dexamethasone (Sigma-Aldrich, St. Louis, MO, USA), and 50 mg/mL ascorbic acid. Differentiation into a neuronal cell lineage was induced by adding neuroglia differentiation medium, which consisted of DMEM/F12 media supplemented with 50 ng/mL NGF, 50 ng/mL BDNF, or 1 mM cyclic adenosine monophosphate (cAMP, Sigma-Aldrich). After 7 d of culture, glial differentiation was assessed by immunofluorescent staining using glial cell markers, such as S100b, glial fibrillary acidic protein (GFAP), and GAP43. The neuronal cell differentiation was estimated by immunofluorescent staining with antibodies against Tuj1, MAP2, and neurofilament (NF200).

### 4.3. Evaluation of Anti-Inflammatory Effects of RvD1 In Vitro and In Vivo

The anti-inflammatory potential of RvD1 was investigated using PNSC spheroids. The spheroids were cultured in ultra-low attachment dishes, seeded in 6-well plates with twenty 50–100 μm spheroids per well, and incubated for 24 h at 37 °C for stabilization. LPS (0.5 μg/mL; Sigma Aldrich, St. Louis, MO, USA) [92,93] was applied for 24 h to induce inflammation. Subsequently, various concentrations (0 nM, 1 nM, 10 nM, and 100 nM) of RvD1 (Cayman Chemical Company, Ann Arbor, MI, USA), dissolved in 1X PBS, were administered to the spheroids. After a 3-day incubation period at 37 °C, the cells were harvested for further analysis.

Proteins were extracted from the collected cells and tissue using RIPA buffer (Thermo Fisher Scientific, Waltham, MA, USA) supplemented with protease and phosphatase inhibitors (GenDEPOT, Katy, TX, USA). These protein samples were then separated by electrophoresis on 10% and 15% SDS-PAGE gels and transferred to PVDF membranes. The membranes were blocked with 5% skim milk for 1.5 h and subsequently incubated overnight at 4 °C with primary antibodies against NF-κB, p65 (1:1000, Santa Cruz Biotechnology, Inc., Dallas, TX, USA), TNF-α (1:1000; Abcam, Cambridge, UK), IL-1 β (1:1000, Abcam), and β-actin (1:4000, Abcam).

Total RNA was extracted from the cells and tissues using TRIzol (Thermo Fisher Scientific). The extracted RNA was then subjected to complementary DNA (cDNA) synthesis following the protocol of cDNA synthesis provided for Platinum Master Mix (GenDEPOT). For each qPCR reaction, 1 μg of RNA was utilized for cDNA synthesis, and 50 ng of cDNA and 200 nM of primer were employed per the manufacturer’s protocol (GenDEPOT). 

### 4.4. Spinal Cord Injury Model and In Vivo Transplantation

Once the PNSCs had grown sufficiently, they were harvested and suspended in PBS for transplantation. Each rat received an injection containing 5 × 10^5^ cells in spheroid form, encapsulated within a 10-μL volume, along with 2 μL of RvD1 (0.1 μg) dissolved in PBS. For the SCI/RvD1+PNSC group, the cells were suspended in an appropriate volume of RvD1 in cell culture media.

The study used Sprague–Dawley rats (200 ± 20 g; OrientBio, Republic of Korea) housed in an animal facility accredited by the Association for Assessment and Accreditation of Laboratory Animal Care at Yonsei University (IACUC2021-0277). All rats were anesthetized with ketamine (100 mg/kg; Yuhan, Seoul, Republic of Korea), Rompun (10 mg/kg; Bayer Korea, Seoul, Republic of Korea), and isopropanol (Hanapharm, Republic of Korea) before surgery. After laminectomy, the spinal cords were exposed and subsequently compressed at the T9 level for 30 s using self-closing forceps. Following surgery, the rats with spinal cord injuries were randomized into five distinct groups: sham (n = 10), SCI/PBS (n = 20), SCI/PNSC spheroid (n = 30), SCI/RvD1 (n = 30), and SCI/RvD1+PNSC (n = 30).

Transplantation of PBS, PNSC spheroids, RvD1, and PNSC+RvD1 was carried out 1 week after injury using a 27-gauge Hamilton syringe at an injection rate of 1 μL/min. To prevent leakage, the needle was left in place for an additional 2 min after injection. Following all surgical procedures, cefazolin (25 mg/kg; Chong Kun Dang, Seoul, Republic of Korea) was administered for 5 days to prevent infection.

### 4.5. Assessment of Post-Injury Motor Behavior Using Open Field Testing

The BBB scale was employed to assess post-injury motor behavior and quantify functional recovery over an 8-week period. This comprehensive tool is used to evaluate rat movements, with the scoring criteria encompassing three-joint movement, weighted stepping, gait coordination, and tail movement. The scale ranges from 0 to 21, with a score of 0 signifying complete paralysis and 21 representing normal movement [4]. The BBB scores were independently assessed by two different evaluators, and the resulting scores were subsequently averaged to obtain a more objective measure of motor recovery. This rigorous methodology ensures the validity of the functional recovery data gathered through open-field testing. 

### 4.6. Assessment of Walking Performance Using Ladder Rung Test

The ladder rung test was employed to assess walking tasks, particularly focusing on limb placement and coordination. The rats were positioned on a transparent-sided ladder with metal rungs and permitted to traverse back and forth twice weekly. Two independent evaluators conducted the assessment, observing and quantifying the occurrences of accurate limb placement out of the total steps taken by each rat. These measurements were subsequently converted into percentage scores, offering an objective evaluation of the rats’ motor coordination and task performance.

### 4.7. Assessment of Mechanical Allodynia

Mechanical allodynia was evaluated between 3 and 4 weeks after SCI, when the rats regained the ability to orient their hind paws downward. The rats were placed in separate compartments of an acrylic container, which was situated on a mesh-like platform. Before stimulation was initiated, the rats were given at least 10 min to acclimate to their surroundings. Stimulation was applied to the hind paws using a dynamic plantar aesthesiometer (Ugo Basile, Comerio, Varese, Italy). A 0.5-mm steel rod was employed to progressively exert pressure on the paws, with the force gradually increasing from 0 to 50 g. The force increment persisted until the rat withdrew its hind paw from the rod. Upon withdrawal, the device automatically recorded both the applied force and the duration of application. This method offered an objective assessment of the mechanical allodynia experienced by the rats following SCI.

### 4.8. Preparation of Tissue Samples

Seven weeks after administration of cells/drugs and 3 weeks post-injection for the cell viability group, the rats underwent perfusion with saline and were subsequently fixed with 4% paraformaldehyde (PFA; Merck, Darmstadt, Germany). Special care was taken to extract spinal cord tissues around the T9 level, which were then fixed in 4% PFA for an additional 24 h at 4 °C. Following this procedure, all samples underwent cryoprotection in 30% sucrose at 4 °C for 1 week. The cryoprotected tissues were then embedded in optimal cutting temperature compound (Sakura Finetek, Torrance, CA, USA) and stored at −80 °C. Tissue sectioning was carried out to generate 20-μm-thick slices for subsequent histological analyses. After staining the tissue with EC staining, the total cord size was measured using Image J software, with the central tissue section serving as the reference. Additionally, the cavity area, myelinated area, and gray matter area were quantified for each tissue sample.

### 4.9. Immunohistochemistry Procedure

The prepared tissue sections were air-dried at room temperature for 30 min and then washed three times with ice-cold 0.3% Tween 20 (Sigma-Aldrich) in PBS. Next, the sections were blocked using a solution containing 10% normal donkey serum, PBS, and 0.3% Triton X-100 (Sigma-Aldrich) for 1 hour at room temperature. The blocked tissue sections were incubated overnight at 4 °C with primary antibodies diluted in the blocking solution. These primary antibodies included mouse anti-CNPase (1:200; Ab6319; Abcam), chicken anti-MAP2 (1:800; Ab5392; Abcam), and goat anti-GFAP (1:800; Ab53554; Abcam).

After incubation, the tissue samples were washed three times with ice-cold 0.3% Tween 20 (Sigma-Aldrich) and then treated with species-specific secondary antibodies, such as DyLight^TM^ 405 donkey anti-mouse immunoglobulin G (IgG, 1:150, Jackson ImmunoResearch, West Grove, PA, USA), CyTM3 donkey anti-chicken immunoglobulin Y (IgG) (H+L) (1:400, Jackson ImmunoResearch), and Alex Fluor^®^ 647 F(ab’)2 fragment donkey anti-goat IgG (H+L) (1:150, Jackson ImmunoResearch), for 1 h at room temperature. The samples were then washed three more times with 0.3% Tween 20 (Sigma-Aldrich) and subsequently cover-slipped using a mounting solution without DAPI (Vector Laboratories, Inc., Burlingame, CA, USA). For the cell viability group, PNSCs initially labeled with green fluorescent protein were mounted using a DAPI-containing mounting solution. Fluorescent images were captured using a confocal laser microscope (LSM700; Carl Zeiss, Oberkochen, Germany).

### 4.10. Eriochrome Cyanine and Cresyl Violet Staining Procedure

Once sectioned, the tissue samples were allowed to air dry at room temperature for several hours. The samples were then immersed in fresh acetone (Daejung Chemicals & Metals Co., Ltd., Republic of Korea) for 5 min, followed by a 15-min period of air drying. The tissues were subsequently incubated in an EC solution (Merck, Kenilworth, NJ, USA) for 30 min at room temperature and then rinsed with distilled water. The following process involved immersing the tissue in 5% iron aluminum (Merck) for 10 min, a crucial step for differentiating and visualizing the gray matter. After this step, the tissues were thoroughly rinsed with distilled water and treated with a borax–ferricyanide solution (Merck) for 3.5 min to ensure complete differentiation. Following another rinse with distilled water, the tissues were briefly stained with cresyl violet (Sigma-Aldrich) for 1 min. The next step involved differentiating the samples using a solution of 95% ethanol with a few drops of 10% acetic acid (Sigma-Aldrich) for 5 min. The tissues were then dehydrated using a graded ethanol series of 95% and 100%. After dehydration, the tissues were cleared with a xylene solution (Daejung Chemicals & Metals Co., Ltd.) and cover-slipped using a permanent mounting medium (Thermo Fisher Scientific). The stained tissue samples were then visualized, and images were captured using a light microscope (IX71; Olympus, Tokyo, Japan). 

### 4.11. Statistical Analysis

All numerical data are presented as the mean value ± the standard error of the mean. Comparative statistical analysis between groups was performed using one-way or two-way analysis of variance (ANOVA) for multiple comparisons. Tukey’s post hoc test was applied for one-way ANOVA, while a mixed-effects model was used for two-way ANOVA. The corresponding *p*-values are denoted as follows: * *p* < 0.05, ** *p* < 0.01, *** *p* < 0.001, and **** *p* < 0.0001. All statistical analyses were conducted using GraphPad PRISM software, version 8.3 (GraphPad Software Inc., San Diego, CA, USA). 

## 5. Conclusions

In our previous research, we found that, when PNSC spheroids were transplanted to the injured site in a spinal cord injury model, they differentiated into neurons and improved neurological function in spinal cord-injured rats with SCI. The neurological function was improved from the initial stage of transplantation, and it was confirmed that the neurological function was remarkably improved at 8 weeks after transplantation. In in vitro and in vivo studies, pro-inflammatory cytokines are greatly increased after spinal cord injury. However, reducing these cytokine secretions proved difficult through the sole transplantation of PNSC spheroids. While RvD1, known for its anti-inflammatory properties, showed some effectiveness in decreasing pro-inflammatory marker expression and macrophage recruitment, the most substantial impact was observed when RvD1 was combined with PNSC spheroids. This combination approach resulted in a reduction in overall macrophage recruitment and an increase in the expression of anti-inflammatory markers. Future research should prioritize strategies that promote cell differentiation, specifically focusing on transplanted PNSC spheroids, to enhance their ability to differentiate into neural cells. In this study, we performed the transplantation of RvD1 1 week after SCI. As a result, it is crucial to further investigate the anti-inflammatory mechanism by which RvD1 inhibits demyelination in the early stages of SCI, aiming to identify potential interventions that could further minimize neuronal damage. 

## Data Availability

All datasets used and/or analyzed during the current study are available from the corresponding author upon reasonable request.

## Abbreviations

SCI	spinal cord injury
PNSC	peripheral nerve-derived stem cell
NCSCs	neural crest stem cells
RvD	resolvin D
qRT-PCR	quantitative reverse transcription polymerase chain reaction
TNF-α	tumor necrosis factor alpha
IL-1β	interleukin-1 beta
IL-1α	interleukin-1 alpha
IL-6	interleukin-6
BM-MSCs	bone marrow-derived mesenchymal stem cells
CB	cord blood
AT	adipose tissue
NSCs	neural stem cells
OPCs	oligodendrocyte progenitor cells
LPS	lipopolysaccharides
NF-κB	nuclear factor kappa-light-chain-enhancer of activated B cells
IL-10	interleukin-10
TGF-β	transforming growth factor beta
BBB	Basso, Beattie, and Bresnahan
PBS	phosphate-buffered saline
EC	eriochrome cyanine
NS	neural stem
NC	neural crest
3D	three-dimensional
GDNF	glial-derived neurotrophic factor
IGF	insulin-like growth factor
NT-3	neurotrophin-3
NSCs	neural stem cells
OECs	olfactory ensheathing cells
SPMs	specialized pro-resolving mediators
AT	aspirin-triggered
PNs	peripheral nerves
DMEM	Dulbecco modified Eagle medium
cDNA	complementary DNA
PFA	paraformaldehyde
IgG	immunoglobulin G
ANOVA	analysis of variance

## References

[1] Scholz J., Finnerup N.B., Attal N., Aziz Q., Baron R., Bennett M.I., Benoliel R., Cohen M., Cruccu G., Davis K.D. (2019). The IASP classification of chronic pain for ICD-11: Chronic neuropathic pain. Pain.

[2] Yang R., Guo L., Wang P., Huang L., Tang Y., Wang W., Chen K., Ye J., Lu C., Wu Y. (2014). Epidemiology of Spinal Cord Injuries and Risk Factors for Complete Injuries in Guangdong, China: A Retrospective Study. PLoS ONE.

[3] Jackson A.B., Dijkers M., DeVivo M.J., Poczatek R.B. (2004). A demographic profile of new traumatic spinal cord injuries: Change and stability over 30 years. Arch. Phys. Med. Rehabil..

[4] Lee H.-L., Yeum C.-E., Lee H., Oh J., Kim J.-T., Lee W.-J., Ha Y., Yang Y.-I., Kim K.-N. (2021). Peripheral Nerve-Derived Stem Cell Spheroids Induce Functional Recovery and Repair after Spinal Cord Injury in Rodents. Int. J. Mol. Sci..

[5] Xiao X., Deng Q., Zeng X., Lai B.-Q., Ma Y.-H., Li G., Zeng Y.-S., Ding Y. (2022). Transcription Profiling of a Revealed the Potential Molecular Mechanism of Governor Vessel Electroacupuncture for Spinal Cord Injury in Rats. Neurospine.

[6] Roolfs L., Hubertus V., Spinnen J., Shopperly L.K., Fehlings M.G., Vajkoczy P. (2022). Therapeutic Approaches Targeting Vascular Repair After Experimental Spinal Cord Injury: A Systematic Review of the Literature. Neurospine.

[7] Bhagwani A., Chopra M., Kumar H. (2022). Spinal Cord Injury Provoked Neuropathic Pain and Spasticity, and Their GABAergic Connection. Neurospine.

[8] Lin A., Shaaya E., Calvert J.S., Parker S.R., Borton D.A., Fridley J.S. (2022). A Review of Functional Restoration from Spinal Cord Stimulation in Patients with Spinal Cord Injury. Neurospine.

[9] Baptiste D.C., Fehlings M.G. (2006). Pharmacological Approaches to Repair the Injured Spinal Cord. J. Neurotrauma.

[10] Jian Y., Sun D., Zhang Z. (2022). A Nomogram Model for Prediction of Tracheostomy in Patients with Traumatic Cervical Spinal Cord Injury. Neurospine.

[11] Kitagawa T., Nagoshi N., Okano H., Nakamura M. (2022). A Narrative Review of Advances in Neural Precursor Cell Transplantation Therapies for Spinal Cord Injury. Neurospine.

[12] Shin H.K., Park J.H., Roh S.W., Jeon S.R. (2022). Meta-Analysis on the Effect of Hypothermia in Acute Spinal Cord Injury. Neurospine.

[13] Bak A.B., Moghaddamjou A., Malvea A., Fehlings M.G. (2022). Impact of Mechanism of Injury on Long-term Neurological Outcomes of Cervical Sensorimotor Complete Acute Traumatic Spinal Cord Injury. Neurospine.

[14] Sekhon L.H., Fehlings M.G. (2001). Epidemiology, Demographics, and Pathophysiology of Acute Spinal Cord Injury. Spine.

[15] Yu D., Mun S.A., Kim S.W., Cho D.-C., Kim C.H., Han I., Lee S., Lee S.-W., Kim K.-T. (2022). Effects of D-Serine and MK-801 on Neuropathic Pain and Functional Recovery in a Rat Model of Spinal Cord Injury. Neurospine.

[16] Rowland J.W., Hawryluk G.W.J., Kwon B., Fehlings M.G. (2008). Current status of acute spinal cord injury pathophysiology and emerging therapies: Promise on the horizon. Neurosurg. Focus.

[17] Eli I., Lerner D.P., Ghogawala Z. (2021). Acute Traumatic Spinal Cord Injury. Neurol. Clin..

[18] Witiw C.D., Fehlings M.G. (2015). Acute Spinal Cord Injury. J. Spinal Disord. Tech..

[19] Lee Y.-S., Kim K.-T., Kwon B.K. (2021). Hemodynamic Management of Acute Spinal Cord Injury: A Literature Review. Neurospine.

[20] Parthiban J., Zileli M., Sharif S.Y. (2020). Outcomes of Spinal Cord Injury: WFNS Spine Committee Recommendations. Neurospine.

[21] Rouanet C., Reges D., Rocha E., Gagliardi V., Silva G.S. (2017). Traumatic spinal cord injury: Current concepts and treatment update. Arq. Neuro-Psiquiatr..

[22] Zavvarian M.-M., Toossi A., Khazaei M., Hong J., Fehlings M. (2020). Novel innovations in cell and gene therapies for spinal cord injury. F1000Research.

[23] Kabu S., Gao Y., Kwon B.K., Labhasetwar V. (2015). Drug delivery, cell-based therapies, and tissue engineering approaches for spinal cord injury. J. Control. Release.

[24] Kettenmann H., Hanisch U.-K., Noda M., Verkhratsky A. (2011). Physiology of Microglia. Physiol. Rev..

[25] Kong X., Gao J. (2017). Macrophage polarization: A key event in the secondary phase of acute spinal cord injury. J. Cell. Mol. Med..

[26] Pineau I., Lacroix S. (2007). Proinflammatory cytokine synthesis in the injured mouse spinal cord: Multiphasic expression pattern and identification of the cell types involved. J. Comp. Neurol..

[27] Oyinbo C.A. (2011). Secondary injury mechanisms in traumatic spinal cord injury: A nugget of this multiply cascade. Acta Neurobiol. Exp..

[28] Gris P., Tighe A., Levin D., Sharma R., Brown A. (2007). Transcriptional regulation of scar gene expression in primary astrocytes. Glia.

[29] Gazdic M., Volarevic V., Harrell C.R., Fellabaum C., Jovicic N., Arsenijevic N., Stojkovic M. (2018). Stem Cells Therapy for Spinal Cord Injury. Int. J. Mol. Sci..

[30] Allison D.J., Ditor D.S. (2015). Immune dysfunction and chronic inflammation following spinal cord injury. Spinal Cord.

[31] David S., López-Vales R., Wee Yong V. (2012). Harmful and beneficial effects of inflammation after spinal cord injury: Potential therapeutic implications. Handb. Clin. Neurol..

[32] Ashammakhi N., Kim H.-J., Ehsanipour A., Bierman R.D., Kaarela O., Xue C., Khademhosseini A., Seidlits S.K. (2019). Regenerative Therapies for Spinal Cord Injury. Tissue Eng. Part B Rev..

[33] Vismara I., Papa S., Rossi F., Forloni G., Veglianese P. (2017). Current Options for Cell Therapy in Spinal Cord Injury. Trends Mol. Med..

[34] Lee S., Nam H., Joo K.-M., Lee S.-H. (2022). Advances in Neural Stem Cell Therapy for Spinal Cord Injury: Safety, Efficacy, and Future Perspectives. Neurospine.

[35] Kim J., Joshi H.P., Sheen S.H., Kim K.-T., Kyung J.W., Choi H., Kim Y.W., Kwon S.Y., Roh E.J., Choi U.Y. (2021). Resolvin D3 Promotes Inflammatory Resolution, Neuroprotection, and Functional Recovery After Spinal Cord Injury. Mol. Neurobiol..

[36] Papa S., Caron I., Rossi F., Veglianese P. (2016). Modulators of microglia: A patent review. Expert Opin. Ther. Pat..

[37] Shechter R., Schwartz M. (2013). Harnessing monocyte-derived macrophages to control central nervous system pathologies: No longer ‘if’ but ‘how’. J. Pathol..

[38] Papa S., Caron I., Erba E., Panini N., De Paola M., Mariani A., Colombo C., Ferrari R., Pozzer D., Zanier E.R. (2016). Early modulation of pro-inflammatory microglia by minocycline loaded nanoparticles confers long lasting protection after spinal cord injury. Biomaterials.

[39] Bisicchia E., Sasso V., Catanzaro G., Leuti A., Besharat Z.M., Chiacchiarini M., Molinari M., Ferretti E., Viscomi M.T., Chiurchiù V. (2018). Resolvin D1 Halts Remote Neuroinflammation and Improves Functional Recovery after Focal Brain Damage Via ALX/FPR2 Receptor-Regulated MicroRNAs. Mol. Neurobiol..

[40] Harrison J.L., Rowe R., Ellis T.W., Yee N.S., O’hara B.F., Adelson P.D., Lifshitz J. (2015). Resolvins AT-D1 and E1 differentially impact functional outcome, post-traumatic sleep, and microglial activation following diffuse brain injury in the mouse. Brain, Behav. Immun..

[41] Gerlach B.D., Marinello M., Heinz J., Rymut N., Sansbury B.E., Riley C.O., Sadhu S., Hosseini Z., Kojima Y., Tang D. (2020). Resolvin D1 promotes the targeting and clearance of necroptotic cells. Cell Death Differ..

[42] Sun L., Wang Y., Wang L., Yao B., Chen T., Li Q., Liu Z., Liu R., Niu Y., Song T. (2019). Resolvin D1 prevents epithelial-mesenchymal transition and reduces the stemness features of hepatocellular carcinoma by inhibiting paracrine of cancer-associated fibroblast-derived COMP. J. Exp. Clin. Cancer Res..

[43] Zakrzewski W., Dobrzyński M., Szymonowicz M., Rybak Z. (2019). Stem cells: Past, present, and future. Stem Cell Res. Ther..

[44] Jiang L., Jones S., Jia X. (2017). Stem Cell Transplantation for Peripheral Nerve Regeneration: Current Options and Opportunities. Int. J. Mol. Sci..

[45] Alessandrini M., Preynat-Seauve O., De Briun K., Pepper M.S. (2019). Stem cell therapy for neurological disorders. S. Afr. Med. J..

[46] Jin M.C., Medress Z.A., Azad T.D., Doulames V.M., Veeravagu A. (2019). Stem cell therapies for acute spinal cord injury in humans: A review. Neurosurg. Focus.

[47] Muheremu A., Peng J., Ao Q. (2016). Stem cell based therapies for spinal cord injury. Tissue Cell.

[48] Penha E.M., Meira C.S., Guimarães E.T., Mendonça M.V.P., Gravely F.A., Pinheiro C.M.B., Pinheiro T.M.B., Barrouin-Melo S.M., Ribeiro-Dos-Santos R., Soares M.B.P. (2014). Use of Autologous Mesenchymal Stem Cells Derived from Bone Marrow for the Treatment of Naturally Injured Spinal Cord in Dogs. Stem Cells Int..

[49] Chen W.-C., Liu W.-F., Bai Y.-Y., Zhou Y.-Y., Zhang Y., Wang C.-M., Lin S., He H.-F. (2021). Transplantation of mesenchymal stem cells for spinal cord injury: A systematic review and network meta-analysis. J. Transl. Med..

[50] Zhu X., Liu Z., Deng W., Zhang Z., Liu Y., Wei L., Zhang Y., Zhou L., Wang Y. (2017). Derivation and characterization of sheep bone marrow-derived mesenchymal stem cells induced with telomerase reverse transcriptase. Saudi J. Biol. Sci..

[51] Seo Y., Shin T.-H., Kim H.-S. (2019). Current Strategies to Enhance Adipose Stem Cell Function: An Update. Int. J. Mol. Sci..

[52] Deng Y.-B., Liu X.-G., Liu Z.-G., Liu X.L., Liu Y., Zhou G.-Q. (2006). Implantation of BM mesenchymal stem cells into injured spinal cord elicits de novo neurogenesis and functional recovery: Evidence from a study in rhesus monkeys. Cytotherapy.

[53] Zurita M., Vaquero J., Bonilla C., Santos M., De Haro J., Oya S., Aguayo C. (2008). Functional Recovery of Chronic Paraplegic Pigs After Autologous Transplantation of Bone Marrow Stromal Cells. Transplantation.

[54] Caron I., Rossi F., Papa S., Aloe R., Sculco M., Mauri E., Sacchetti A., Erba E., Panini N., Parazzi V. (2016). A new three dimensional biomimetic hydrogel to deliver factors secreted by human mesenchymal stem cells in spinal cord injury. Biomaterials.

[55] Kuh S.-U., Cho Y.-E., Yoon D.-H., Kim K.-N., Ha Y. (2005). Functional recovery after human umbilical cord blood cells transplantation with brain-derived neutrophic factor into the spinal cord injured rat. Acta Neurochir..

[56] Chua S.J.M., Bielecki R.D., Yamanaka N.M., Fehlings M.G.M., Rogers I.M., Casper R.F.M. (2010). The Effect of Umbilical Cord Blood Cells on Outcomes After Experimental Traumatic Spinal Cord Injury. Spine.

[57] Yao L., He C., Zhao Y., Wang J., Tang M., Li J., Wu Y., Ao L., Hu X. (2013). Human umbilical cord blood stem cell transplantation for the treatment of chronic spinal cord injury: Electrophysiological changes and long-term efficacy. Neural Regen. Res..

[58] Zuk P.A., Zhu M.I., Mizuno H., Huang J., Futrell J.W., Katz A.J., Benhaim P., Lorenz H.P., Hedrick M.H. (2001). Multilineage Cells from Human Adipose Tissue: Implications for Cell-Based Therapies. Tissue Eng..

[59] Ahmadian Kia N., Bahrami A.R., Ebrahimi M., Matin M.M., Neshati Z., Almohaddesin M.R., Aghdami N., Bidkhori H.R. (2011). Comparative Analysis of Chemokine Receptor’s Expression in Mesenchymal Stem Cells Derived from Human Bone Marrow and Adipose Tissue. J. Mol. Neurosci..

[60] Lee R.H., Kim B., Choi I., Kim H., Choi H.S., Suh K., Bae Y.C., Jung J.S. (2004). Characterization and Expression Analysis of Mesenchymal Stem Cells from Human Bone Marrow and Adipose Tissue. Cell. Physiol. Biochem..

[61] Planat-Benard V., Silvestre J.-S., Cousin B., André M., Nibbelink M., Tamarat R., Clergue M., Manneville C., Saillan-Barreau C., Duriez M. (2004). Plasticity of Human Adipose Lineage Cells Toward Endothelial Cells: Physiological and therapeutic perspectives. Circulation.

[62] Safford K.M., Hicok K.C., Safford S.D., Halvorsen Y.D., Wilkison W.O., Gimble J.M., Rice H.E. (2002). Neurogenic differentiation of murine and human adipose-derived stromal cells. Biochem. Biophys. Res. Commun..

[63] Ohta Y., Hamaguchi A., Ootaki M., Watanabe M., Takeba Y., Iiri T., Matsumoto N., Takenaga M. (2017). Intravenous infusion of adipose-derived stem/stromal cells improves functional recovery of rats with spinal cord injury. Cytotherapy.

[64] Emgård M., Piao J., Aineskog H., Liu J., Calzarossa C., Odeberg J., Holmberg L., Samuelsson E.-B., Bezubik B., Vincent P.H. (2014). Neuroprotective effects of human spinal cord-derived neural precursor cells after transplantation to the injured spinal cord. Exp. Neurol..

[65] Yousefifard M., Rahimi-Movaghar V., Nasirinezhad F., Baikpour M., Safari S., Saadat S., Moghadas Jafari A., Asady H., Razavi Tousi S.M., Hosseini M. (2016). Neural stem/progenitor cell transplantation for spinal cord injury treatment; A systematic review and meta-analysis. Neuroscience.

[66] Zhao Y., Zuo Y., Jiang J., Yan H., Wang X., Huo H., Xiao Y. (2016). Neural stem cell transplantation combined with erythropoietin for the treatment of spinal cord injury in rats. Exp. Ther. Med..

[67] Hawryluk G.W., Mothe A., Wang J., Wang S., Tator C., Fehlings M.G. (2012). An In Vivo Characterization of Trophic Factor Production Following Neural Precursor Cell or Bone Marrow Stromal Cell Transplantation for Spinal Cord Injury. Stem Cells Dev..

[68] Joseph N.M., Mukouyama Y.-S., Mosher J.T., Jaegle M., Crone S.A., Dormand E.-L., Lee K.-F., Meijer D., Anderson D.J., Morrison S.J. (2004). Neural crest stem cells undergo multilineage differentiation in developing peripheral nerves to generate endoneurial fibroblasts in addition to Schwann cells. Development.

[69] Liu J.A., Cheung M. (2016). Neural crest stem cells and their potential therapeutic applications. Dev. Biol..

[70] Mehrotra P., Tseropoulos G., Bronner M.E., Andreadis S.T. (2020). Adult tissue–derived neural crest-like stem cells: Sources, regulatory networks, and translational potential. STEM CELLS Transl. Med..

[71] Nakhjavan-Shahraki B., Yousefifard M., Rahimi-Movaghar V., Baikpour M., Nasirinezhad F., Safari S., Yaseri M., Moghadas Jafari A., Ghelichkhani P., Tafakhori A. (2018). Transplantation of olfactory ensheathing cells on functional recovery and neuropathic pain after spinal cord injury; systematic review and meta-analysis. Sci. Rep..

[72] Denaro S., D’aprile S., Alberghina C., Pavone A.M., Torrisi F., Giallongo S., Longhitano L., Mannino G., Furno D.L., Zappalà A. (2022). Neurotrophic and immunomodulatory effects of olfactory ensheathing cells as a strategy for neuroprotection and regeneration. Front. Immunol..

[73] Gómez R.M., Sánchez M.Y., Portela-Lomba M., Ghotme K., Barreto G.E., Sierra J., Moreno-Flores M.T. (2018). Cell therapy for spinal cord injury with olfactory ensheathing glia cells (OECs). Glia.

[74] Yang H., He B.-R., Hao D.-J. (2015). Biological Roles of Olfactory Ensheathing Cells in Facilitating Neural Regeneration: A Systematic Review. Mol. Neurobiol..

[75] Roohbakhsh A., Etemad L., Karimi G. (2022). Resolvin D1: A key endogenous inhibitor of neuroinflammation. Biofactors.

[76] Laste G., Ripoll Rozisky J., de Macedo I.C., Souza Dos Santos V., Custodio de Souza I.C., Caumo W., Torres I.L. (2013). Spinal Cord Brain-Derived Neurotrophic Factor Levels Increase after Dexamethasone Treatment in Male Rats with Chronic Inflammation. Neuroimmunomodulation.

[77] Chen X., Ma L., Jiang Y., Chen S., Zhu C., Liu M., Ma X., Zhu D., Liu Y., Peng F. (2012). Minocycline up-regulates the expression of brain-derived neurotrophic factor and nerve growth factor in experimental autoimmune encephalomyelitis. Eur. J. Pharmacol..

[78] Kigerl K.A., Gensel J.C., Ankeny D.P., Alexander J.K., Donnelly D.J., Popovich P.G. (2009). Identification of Two Distinct Macrophage Subsets with Divergent Effects Causing either Neurotoxicity or Regeneration in the Injured Mouse Spinal Cord. J. Neurosci..

[79] Levi M., Brimble M.A. (2004). A Review of Neuroprotective Agents. Curr. Med. Chem..

[80] Yousof S.M., ElSayed D.A., El-Baz A.A., Sallam H.S., Abbas F. (2021). Combined Treatment of Adipose Derived-Mesenchymal Stem Cells and Pregabalin Is Superior to Monotherapy for the Treatment of Neuropathic Pain in Rats. Stem Cells Int..

[81] Yin P., Wei Y., Wang X., Zhu M., Feng J. (2018). Roles of Specialized Pro-Resolving Lipid Mediators in Cerebral Ischemia Reperfusion Injury. Front. Neurol..

[82] Recchiuti A., Isopi E., Romano M., Mattoscio D. (2020). Roles of Specialized Pro-Resolving Lipid Mediators in Autophagy and Inflammation. Int. J. Mol. Sci..

[83] Sulciner M.L., Serhan C.N., Gilligan M.M., Mudge D.K., Chang J., Gartung A., Lehner K.A., Bielenberg D.R., Schmidt B., Dalli J. (2018). Resolvins suppress tumor growth and enhance cancer therapy. J. Exp. Med..

[84] Park J., Langmead C.J., Riddy D.M. (2020). New Advances in Targeting the Resolution of Inflammation: Implications for Specialized Pro-Resolving Mediator GPCR Drug Discovery. ACS Pharmacol. Transl. Sci..

[85] Xu Z.-Z., Berta T., Ji R.-R. (2013). Resolvin E1 Inhibits Neuropathic Pain and Spinal Cord Microglial Activation Following Peripheral Nerve Injury. J. Neuroimmune Pharmacol..

[86] Park C.-K., Xu Z.-Z., Liu T., Lü N., Serhan C.N., Ji R.-R. (2011). Resolvin D2 Is a Potent Endogenous Inhibitor for Transient Receptor Potential Subtype V1/A1, Inflammatory Pain, and Spinal Cord Synaptic Plasticity in Mice: Distinct Roles of Resolvin D1, D2, and E1. J. Neurosci..

[87] Zhang L., Terrando N., Xu Z.-Z., Bang S., Jordt S.-E., Maixner W., Serhan C.N., Ji R.-R. (2018). Distinct Analgesic Actions of DHA and DHA-Derived Specialized Pro-Resolving Mediators on Post-operative Pain After Bone Fracture in Mice. Front. Pharmacol..

[88] Gemperle C., Tran S., Schmid M., Rimann N., Marti-Jaun J., Hartling I., Wawrzyniak P., Hersberger M. (2021). Resolvin D1 reduces inflammation in co-cultures of primary human macrophages and adipocytes by triggering macrophages. Prostaglandins Leukot. Essent. Fat. Acids.

[89] Serhan C.N., Chiang N., Dalli J., Levy B.D. (2014). Lipid Mediators in the Resolution of Inflammation. Cold Spring Harb. Perspect. Biol..

[90] Wang C.-S., Maruyama C.L., Easley J.T., Trump B.G., Baker O.J. (2017). AT-RvD1 Promotes Resolution of Inflammation in NOD/ShiLtJ mice. Sci. Rep..

[91] Xiang S.-Y., Ye Y., Yang Q., Xu H.R., Shen C.-X., Ma M.-Q., Jin S.-W., Mei H.-X., Zheng S.-X., Smith F.-G. (2021). RvD1 accelerates the resolution of inflammation by promoting apoptosis of the recruited macrophages via the ALX/FasL-FasR/caspase-3 signaling pathway. Cell Death Discov..

[92] Dai W., Wang M., Wang P., Wen J., Wang J., Cha S., Xiao X., He Y., Shu R., Bai D. (2021). lncRNA NEAT1 ameliorates LPS-induced inflammation in MG63 cells by activating autophagy and suppressing the NLRP3 inflammasome. Int. J. Mol. Med..

[93] Krejcova D., Pekarova M., Safrankova B., Kubala L. (2009). The effect of different molecular weight hyaluronan on macrophage physiology. Neuro Endocrinol. Lett..

